# Ayurvedic vs. Conventional Nutritional Therapy Including Low-FODMAP Diet for Patients With Irritable Bowel Syndrome—A Randomized Controlled Trial

**DOI:** 10.3389/fmed.2021.622029

**Published:** 2021-09-06

**Authors:** Michael Jeitler, Till Wottke, Dania Schumann, Laura M. Puerto Valencia, Andreas Michalsen, Nico Steckhan, Martin Mittwede, Elmar Stapelfeldt, Daniela Koppold-Liebscher, Holger Cramer, Manfred Wischnewsky, Vijayendra Murthy, Christian S. Kessler

**Affiliations:** ^1^Institute of Social Medicine, Epidemiology and Health Economics, Charité – Universitätsmedizin Berlin, Corporate Member of Freie Universität Berlin and Humboldt-Universität zu Berlin, Berlin, Germany; ^2^Department of Internal and Integrative Medicine, Immanuel Hospital Berlin, Berlin, Germany; ^3^Digital Health Center, Hasso Plattner Institute, University of Potsdam, Potsdam, Germany; ^4^Department of Religious Studies, Goethe-University, Frankfurt am Main, Germany; ^5^Department of Internal and Integrative Medicine, University of Duisburg-Essen, Evang. Kliniken Essen-Mitte, Essen, Germany; ^6^Department of Mathematics and Computer Science, University Bremen, Bremen, Germany; ^7^College of Medicine, London, United Kingdom; ^8^Australian Research Centre in Complementary and Integrative Medicine, Faculty of Health, University of Technology, Sydney, NSW, Australia

**Keywords:** nutrition – clinical, Traditional Indian Medicine, irritable bowel syndrome, Ayurveda, clinical trials, complementary medicine, integrative medicine

## Abstract

**Aims:** To compare the effects of Ayurvedic and conventional nutritional therapy in patients with irritable bowel syndrome (IBS).

**Methods:** Sixty-nine patients with IBS were randomized to Ayurvedic (*n* = 35) or conventional nutritional therapy according to the recommendations of the German Nutrition Society including the low-FODMAP diet (*n* = 34). Study visits took place at baseline and after 1, 3, and 6 months. The primary outcome was IBS symptom severity (IBS-SSS) after 3 months; secondary outcomes included stress (CPSS), anxiety and depression (HADS), well-being (WHO-5) and IBS-specific quality of life (IBS-QOL). A repeated measures general linear model (GLM) for intent-to-treat-analyses was applied in this explorative study.

**Results:** After 3 months, estimated marginal means for IBS-SSS reductions were 123.8 [95% confidence interval (95% CI) = 92.8–154.9; *p* < 0.001] in the Ayurvedic and 72.7 (95% CI = 38.8–106.7; *p* < 0.001) in the conventional group. The IBS-SSS reduction was significantly higher in the Ayurveda group compared to the conventional therapy group (estimated marginal mean = 51.1; 95% CI = 3.8–98.5; *p* = 0.035) and clinically meaningful. Sixty-eight percentage of the variance in IBS-SSS reduction after 3 months can be explained by treatment, 6.5% by patients' expectations for their therapies and 23.4% by IBS-SSS at pre-intervention. Both therapies are equivalent in their contribution to the outcome variance. The higher the IBS-SSS score at pre-intervention and the larger the patients' expectations, the greater the IBS-SSS reduction. There were no significant group differences in any secondary outcome measures. No serious adverse events occurred in either group.

**Conclusion:** Patients with IBS seem to benefit significantly from Ayurvedic or conventional nutritional therapy. The results warrant further studies with longer-term follow-ups and larger sample sizes.

**Clinical Trial Registration:**https://clinicaltrials.gov/ct2/show/NCT03019861, identifier: NCT03019861.

## Introduction

Irritable bowel syndrome (IBS) is one of the most common gastrointestinal disorders with a prevalence between 5 and 10% for many European countries and the USA (1). Both the individual burden of typical clinical symptoms and the discussed pathophysiological mechanisms may differ from patient to patient and IBS subtypes (2). A coherent link between certain pathologies and the symptoms of IBS is still unclear. In addition, IBS is often associated with other somatic comorbidities and mental disorders (3). IBS has considerable consequences in terms of quality of life, work productivity and burden on health-care systems (4).

Dietary changes are one of the most commonly used interventions in patients with IBS (5), especially the low fermentable oligo-, di-, monosaccharides and polyols (FODMAP)-diet, which showed clinically meaningful responses in 50–86% of patients (6).

Some patients with IBS report dissatisfaction with conventional medical therapies and seek other forms of treatment especially Complementary and Integrative Medicine (CIM) (2). Of the various CIM interventions, the Traditional Indian Medicine Ayurveda, a Whole Medical System, is increasingly used worldwide and is recognized by the World Health Organization as a medical science (7). In Europe and the United States, Ayurveda has become increasingly popular in recent years, especially in the treatment of chronic and psychosomatic disorders (8).

Ayurvedic IBS-treatment is based on diagnosing the condition from an Ayurvedic perspective, which takes into consideration inter-person variablity of digestive functions, physio-psychological personality type and variations in the presenting symptoms (9). The treatment approach in Ayurveda is thereby a customized nutritional therapy tailored to the individual constellation of various factors of the patient including their symptoms, constitution, digestive capacity, bowel sensitivity, composition of the *milieu intérieur*, individual food habits assessed during the diagnostic processes and the like (10, 11). Ayurvedic diets are fairly easy to implement by patients in their daily lives and generally are comparatively inexpensive methods of self-care. In Ayurveda, customized nutritional therapy is most often used to treat patients with IBS. Additionally, oral herbal preparations and/or specific types of Ayurvedic enema therapies are used in case of therapeutic resistance to nutritional therapy (11). However, to date, little systematic data is available on the effectiveness of Ayurvedic nutritional therapy in comparison to conventional nutritional therapy for IBS patients and the feasibility of such an approach in western settings.

Therefore, the primary objective of this non-inferiority study was to test whether an Ayurvedic nutritional therapy provides at least comparable benefits to the patients as a conventional nutritional therapy according to the recommendations of the German Nutrition Society (DGE) including the low-FODMAP diet on IBS symptom severity in patients with IBS (in mathematical terms: Ayurvedic nutritional therapy ≥ conventional nutritional therapy). This includes that Ayurvedic nutritional therapy can also be better than conventional nutritional therapy. The hypotheses as in most clinical trials, were stated in terms of differences in the mean response of an outcome of interest, here IBS-SSS reduction (IBS-SSS at pre-intervention – after 3 months) and adjusted for various covariates.

## Methods

### Study Design

In a two-armed multicenter randomized controlled clinical trial, IBS patients were allocated 1:1 to two treatment groups: (1) Ayurvedic nutritional therapy and (2) conventional nutritional therapy according to the recommendations of the DGE including low-FODMAP diet. The study protocol was approved by the ethics committee of the Charité – Universitätsmedizin Berlin (EA4/115/16) and the University Hospital Essen (16-7254-BO) and all participants gave their informed consent. The trial was registered at ClinicalTrials.gov prior to patient recruitment (NCT03019861). Trained study personnel performed collection of data at Immanuel Hospital Berlin, Department of Internal and Integrative Medicine, Berlin, Germany and at Evang. Kliniken Essen-Mitte, Department of Internal and Integrative Medicine, Essen, Germany.

### Participants

Volunteers, who lived in the region of Berlin and Essen, were recruited by local newspaper advertisements and flyers. Subjects were included if they (1) were aged 18–70 years of all sexes, and (2) had the diagnosis IBS according to the the German S3 IBS guideline (12, 13), diagnosed by an external physician. Subjects were excluded if they (1) had a generally poor overall state of health, (2) had a serious acute or chronic co-morbidity, (3) were pregnant or breast feeding, (4) had a pre-diagnosed eating disorder, (5) were in recognition procedure for early retirement or disability, and/or (6) participated in another clinical trial.

### Outcome Measures

All participants were asked to complete standardized validated questionnaires at the beginning of the study, at 1 month, at 3 months and at 6 months follow-up. The primary outcome was change of the mean score of the German version of Irritable Bowel Syndrome – Severity Scoring System (IBS-SSS) questionnaire at 3 months (14). We modified the visual analog scales in the original IBS-SSS version to 4 (question no. 3 and 4), respectively, 5-point (question no. 1b and 2b) Likert scales, since all questionnaires were filled out online in Limesurvey. Pre-specified secondary outcomes included the following validated questionnaires in German:

- Irritable Bowel Syndrome – Quality Of Life (IBS-QOL), a 34-item scale designed for the assessment of quality of life in patients with IBS (15).
- Quality of life and well-being were assessed by the 5-item WHO-Five Well-being index (WHO-5) (16).
- Cohen Perceived Stress Scale (CPSS), a 10-item scale for measuring personal levels of experienced and perceived stress (17).
- Hospital Anxiety and Depression Scale (HADS), a 14-item scale designed for the assessment of anxiety and depression symptoms in general populations (18).

### Additional Parameters

Adverse events were systematically ascertained. Moreover, additional questions on 5-point Likert scales at month 3 (1: agree fully to 5: disagree fully) and evaluation questions at month 6 were asked, using Numeric Rating Scale (NRS) questions regarding adherence to diet and health (0: not at all to 10: very). Adherence to diet was assessed by the patients themselves and through an external assessment by dieticians based on dietary protocols and records made during the consultations (NRS 0: not at all to 10: very).

Furthermore, microbiome analysis and qualitative focus group interviews were conducted, results of which will be published elsewhere.

### Randomization and Masking

After completing the baseline questionnaires, the participants were randomly assigned to one of the study arms. Block randomization (block-size 4) was used. The randomization list was created by the biometrician not involved in patient recruitment or assessment based on the Blockrand-package (Version 1.3) in R. The list was password-secured and no other person than the biometrician was able to access it. The subjects were randomly allocated by opening of a sealed envelope prepared by a study nurse not involved in the study. As with all therapy trials, the participants, therapists and research assistants who assisted in the therapy arrangements could not be blinded regarding the treatment allocation. Also the study directors and statisticians conducting outcome analysis were not masked.

### Interventions

With each participant, patient history was taken, and all participants received a 45 ± 10 min. personal nutritional therapy session (baseline consultation), followed by two further 30 ± 10 min. nutritional therapy sessions 3 and 8 weeks after the baseline consultation. The study was conducted in comparable German outpatient clinic settings in Berlin and Essen. The basic principles for the interventions were defined by a consensus process prior to enrolment of the study participants.

The Ayurvedic approach included individualized nutritional recommendations for a diet based on Ayurvedic concepts, predominantly on the concept of strength of digestion and metabolism (“digestive fire,” Sanskrit: = *agni*), which is further referred to in this paper as “general nutritional therapy of Ayurveda” (11, 19). Customized advice was dependent on individual symptoms and circumstances (“specific nutritional therapy of Ayurveda”) (9, 20). The main content of both therapeutic aspects are summarized in the Supplementary Material *General and Specific Nutritional Therapy of Ayurveda*. Specific suggestions and details for food items, recipes, food preparation, timings, spices etc. were given to each participant and the list of food was adapted to local availability in order to maximize practicability and participants' adherence to the intervention. Two experienced Ayurveda nutritional experts and registered German naturopaths (German: “*Heilpraktiker*”) with each more than 500 h of academic training in Ayurveda and more than 10 years of continuous clinical experience with Ayurveda in Germany performed the counseling sessions in both study centers.

Conventional treatment consisted of nutritional therapy in accordance with the German Nutrition Society (Deutsche Gesellschaft für Ernährung, DGE) with specific suggestions for foods and recipes (21). Two experienced German dieticians with more than 3 years of continuous clinical experience in treating patients performed the nutritional therapy. Participants also received a brochure with nutritional recommendations for IBS patients, which was prepared by the DGE (21). This brochure explained the principles of a balanced diet and the essence of a low-FODMAP diet. In particular, participants were informed that foods rich in fructans and galacto-oligosaccharides (e.g., wheat, rye, onions, and legumes), as well as items containing lactose, foods with an excess of fructose (e.g., apples, mangoes, and honey), and foods rich in sorbitol, mannitol, maltitol, and xylitol (e.g., apricots, peaches) should be avoided. To help participants select suitable foods, they were also given a series of low-FODMAP recipes, a list of foods they should avoid, and another list of foods they could eat instead. After the 12-week intervention period of the study (elimination phase), participants re-examined a different FODMAP group each week for 2–3 days per week to test individual tolerance to each of the FODMAP groups (reintroduction phase).

All participants were asked to maintain their routine activities and not to begin any other treatment during the study.

### Statistical Analysis

Group sample sizes of 36 and 36 achieve 80% power to detect non-inferiority using a one-sided, two-sample *t*-test in the present explorative study. The primary objective was to estimate the therapeutic quality and difference of both therapies. The significance level (alpha) of the test is 0.05. The margin of equivalence is 50 points, which is the minimally clinically important difference (MID), i.e., that “patients perceive as important, either beneficial or harmful, and that would lead the clinician to consider a change in the patient's management” (22). The null and the alternative hypothesis of this non-inferiority clinical trial are H_0_: μ_1_ – μ_0_ ≤ -δ vs. H_1_: μ_1_ – μ_0_ > -δ, where δ ≥ 0. δ is called the margin of clinical significance which is in our case 50 (=MID). The true difference between the Ayurveda mean and the conventional nutritional therapy mean is assumed to be 35 points. The estimates of the standard deviations are assumed to be 25 points for both groups (22).

This intention-to-treat (ITT) randomized study was designed to test whether or not the mean effectiveness [as measured by the primary outcome parameter IBS-SSS reduction (IBS-SSS at pre-intervention – after 3 months) and adjusted for various covariates] of the Ayurvedic nutritional therapy is non-inferior to the mean effectiveness of the conventional nutritional therapy. Missing data were handled by maximum likelihood multiple imputation. Additional parameters (section Additional Parameters) were not imputed. Generalized Linear Models (GLM) were primarily used to reduce within-group error variance and to eliminate confounding factors. For checking the assumptions for GLMs we carried out Levene's test, for homogeneity of variances, Shapiro-Wilk's resp. Kolmogorov's test for normality with Lilliefors significance correction and the test for homogeneity of regression slopes. The estimated marginal means section of the output gives the adjusted means (controlling for the covariates) for each diet group. For *post-hoc* comparisons of the main effects we used a Sidak correction for confidence interval adjustment. We included study centers as a random effect, treatment group as a fixed factor, IBS-SSS at pre-intervention and for sensitivity analysis participants' expectations for their individual therapy at pre-intervention as covariates. All statistical analyses were done within the statistical programming language R (Version 3.5.2), SPSS (Version 26; IBM) and NCSS (Version 10). All authors had access to the study data and reviewed and approved the final manuscript.

## Results

**Figure 1:**
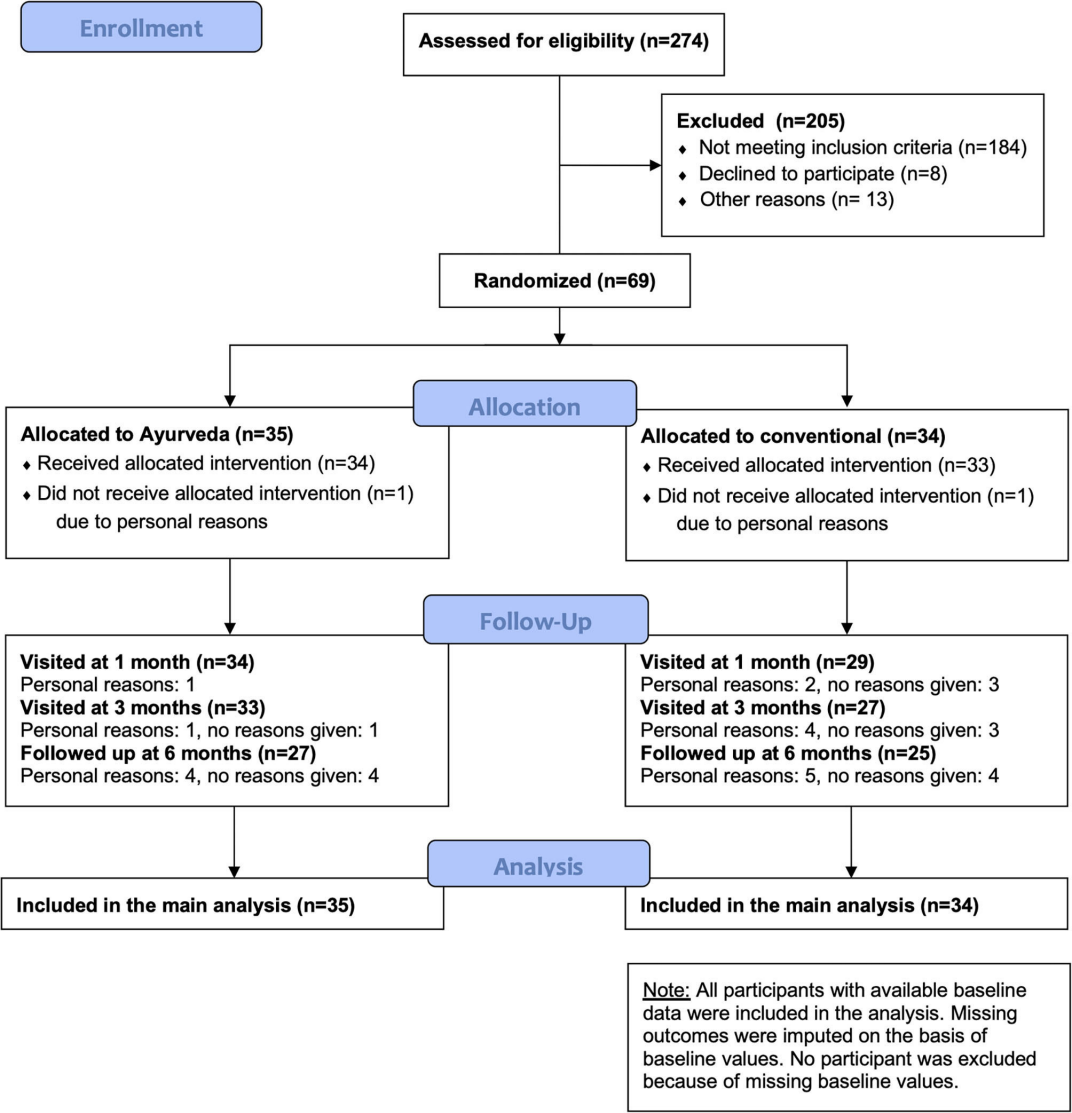
Flowchart.

A total of 274 patients were screened for eligibility (Figure 1). Most patients were excluded because of other gastrointestinal diagnoses and consequently did not have a diagnosis of IBS. Sixty-nine patients fulfilled the eligibility criteria and were enrolled into the study (34 in the conventional group and 35 in the Ayurveda group). The first patient was enrolled in January 2017; follow-up visits for the last patient were completed by February 2019. Overall, 60 participants (87%) completed the visit at 3 months, 52 (75%) completed the follow up at 6 months. Sixty-nine data sets were included in the final analysis.

### Baseline Characteristics

Mean patients age in the Ayurveda group (*n* = 35) was 50.8 ± 12.0 years, in the conventional group (*n* = 34) 41.8 ± 14.4 years. Eighty percent of the patients in the Ayurveda group and 71% in the conventional group were women (Table 1). The difference of IBS-SSS at pre-intervention between both therapy groups was 6.5 ± 18.7 points. Duration of IBS diagnosis was 9.2 ± 10.0 years in the Ayurveda group and 6.7 ± 7.3 years in the conventional group. Patients' expectations for Ayurvedic therapy as well as for conventional therapy did not significantly differ between both therapy groups (Table 1). Overall patients with Ayurvedic nutritional treatment had a significantly (*p* = 0.003) higher mean expectation at pre-intervention for the benefit of their treatment (7.4 ± 2.4) compared to the corresponding patients with conventional therapy (5.7 ± 2.1). We could not find random imbalances in prognostic factors which may otherwise bias intention-to-treat effect.

**Table 1:** Baseline characteristics.

		**Treatment groups**		**Sig.** ^*^
	**Total**	**Conventional nutritional** **therapy**	**Ayurvedic nutritional** **therapy**	
	**(*n* = 69)**	**(*n* = 34)**	**(*n* = 35)**	
Age	46.4 ± 13.9	41.8 ± 14.4	50.8 ± 12.0	0.006
	Median: 50	Median: 38	Median: 52	
Sex (women)	75.4%	70.6%	80.0%	0.364
Body mass index	24.1 ± 3.7	23.3 ± 3.5	25.9 ± 3.7	0.052
Duration of IBS diagnosis (years)	8.0 ± 8.8	6.7 ± 7.3	9.2 ± 10.0	0.264
Patients' expectations for conventional nutritional therapy	5.7 ± 2.3	5.7 ± 2.1	5.6 ± 2.4	0.171
Patients' expectations for Ayurvedic nutritional therapy	7.4 ± 2.0	7.5 ± 1.6	7.4 ± 2.3	0.072
Previous drug therapy for IBS				
Analgetics	3 (4.4%)	1 (3.0%)	2 (5.7%)	0.59
Antispasmodics	5 (7.4%)	1 (3.0%)	4 (11.4%)	0.19
Laxatives	2 (2.9%)	1 (3.0%)	1 (2.9%)	0.97
Antidiarrheal drugs	4 (5.9%)	1 (3.0%)	3 (8.6%)	0.33
Probiotics	28 (41.2%)	14 (42.4%)	14 (40.0%)	0.84
Phytotherapeutic agents	27 (39.7%)	14 (42.4%)	13 (37.1%)	0.66
Concomitant and previous illnesses				
Cardiovascular diseases	16 (23.5%)	6 (18.2%)	10 (28.6%)	0.42
Renal diseases	4 (5.9%)	3 (9.1%)	1 (2.9%)	0.24
Metabolic diseases	8 (11.8%)	1 (3.0%)	7 (20.0%)	0.40
Skin diseases	5 (7.4%)	3 (9.1%)	2 (5.7%)	0.17
Irritable Bowel Syndrome – Severity Scoring System (IBS-SSS)	275.9 ± 77.1	279 ± 78.2	272.7 ± 77.1	0.731
Quality Of Life (IBS-QOL)	53.0 ± 20.7	52.6 ± 21.8	53.4 ± 19.9	0.862
Cohen Perceived Stress Scale (CPSS)	19.7 ± 7.0	19.4 ± 7.7	19.9 ± 6.3	0.767
Hospital Anxiety and Depression Scale (HADS-Total)	14.1 ± 6.0	13.7 ± 6.2	14.6 ± 5.8	0.524
Hospital Anxiety Scale (HADS-A)	8.3 ± 3.7	7.9 ± 3.9	8.6 ± 3.6	0.447
Hospital Depression Scale (HADS-D)	5.8 ± 3.3	5.7 ± 3.6	5.9 ± 3.0	0.767
WHO-5 Well-Being Index	43.7 ± 20.5	45.1 ± 20.2	42.4 ± 20.9	0.594

* *Statistical tests to compare baseline data in randomized controlled trials have remained questionable since 1990 (Ahn, 2019). Tests of baseline differences are not necessarily wrong, just illogical (Senn, 1994)*.

### Primary Outcome

After 3 months, the mean values of IBS-SSS were reduced from 272.7 ± 77.1 to 166.9 ± 92.0 in the Ayurveda group and from 279.2 ± 78.2 to 199.7 ± 98.3 in the conventional group. The means of the paired differences between IBS-SSS at baseline and after 3 months were 105.9 ± 83.8 [95% confidence interval (95% CI) = 77.1–134.7; *p* < 0.001] for Ayurvedic nutritional therapy and 79.5 ± 126.0 (95% CI = 35.6–123.5; *p* = 0.001) for conventional therapy.

The assumptions of equality of error variances for IBS-SSS reduction after 3 months was not violated (Levene's test *p* = 0.152). The estimated marginal means (also known as adjusted means or predicted means) for IBS-SSS reduction after 3 months (controlled for patients' expectations for their therapies and IBS-SSS at pre-intervention as covariates and centers as random factor) were 123.8 ± 15.5 (95% CI = 92.8–154.9) in the Ayurveda group and 72.7 ± 17.0 (95% CI = 38.8–106.7) in the conventional group. The mean difference of the IBS-SSS reductions between both therapy groups based on the estimated marginal means was statistically significant in favor of the Ayurveda group (mean difference 51.1 ± 23.7; 95% CI = 3.8–98.5; *p* = 0.035). The mean difference of 51.1 was above the minimally clinically important difference (MID) of IBS-SSS (MID = 50) (23) and thus beside statistically significant also clinically significant.

Tests of “between-subjects effects” showed that the effect size of the treatment was large (partial η^2^ = 0.68; Cohen's f = 1.46). Sixty-eight percentage of the variance in IBS-SSS reduction after 3 months can be explained by the variable treatment. Furthermore, we found in this model no significant difference in IBS-SSS reduction after 3 months between both treatments (*p* = 0.343). In contrast to this result, patients' expectations for their therapies and IBS-SSS at pre-intervention both had a significant influence on outcome [expectations: *p* = 0.041; *F*_(df1, error63)_ = 4.4; partial η^2^ = 0.065; IBS-SSS at pre-intervention: *p* < 0.001; *F*_(df1, error1.02)_ = 19.3; partial η^2^ = 0.234]. The variable centers was not significant (*p* = 0.454). 23.4% of the variance in IBS-SSS reduction after 3 months can be explained by IBS-SSS at pre-intervention and 6.5% by patients' expectations for their therapies at pre-intervention.

**Figure 2:**
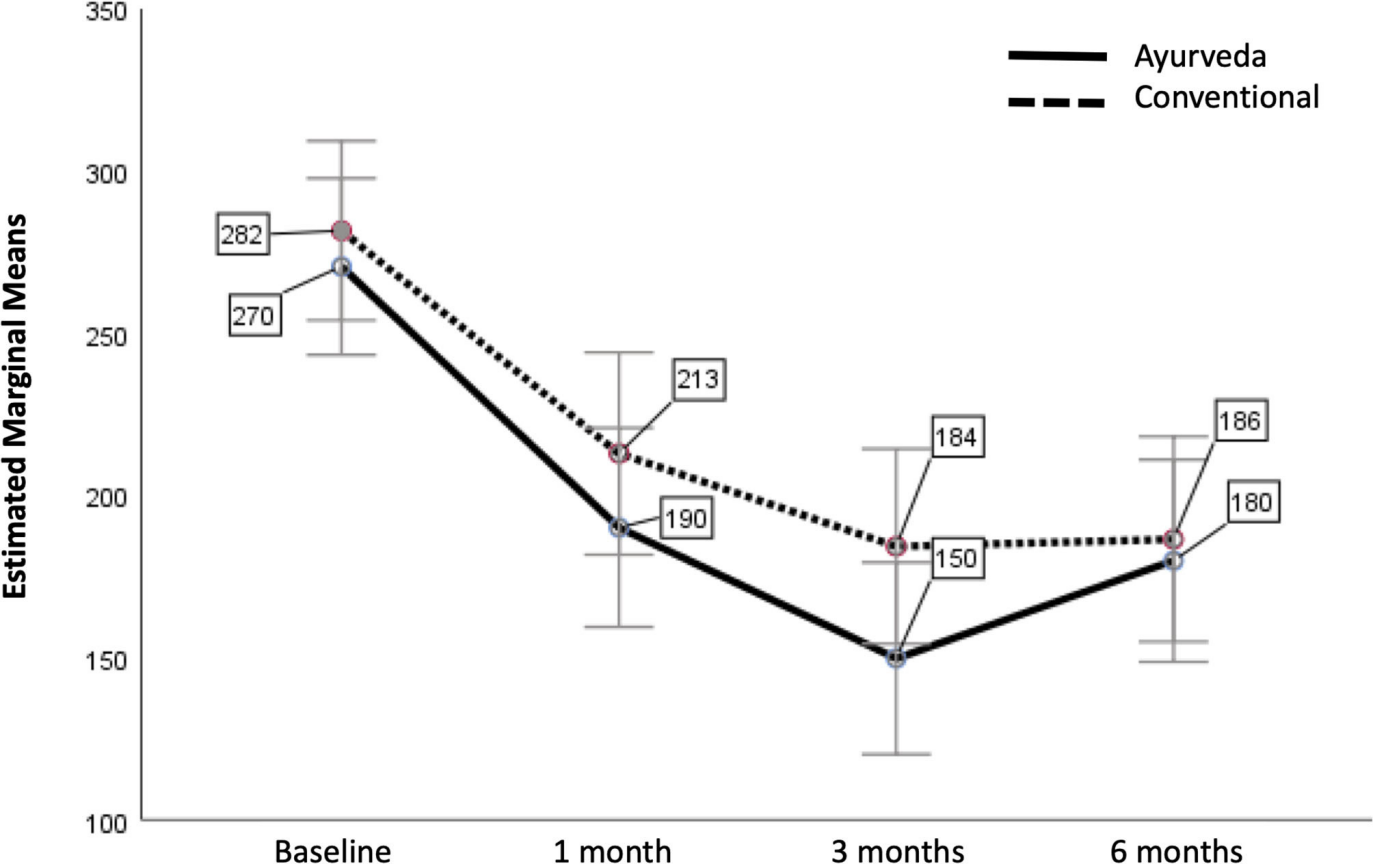
Estimated Marginal Means of IBS-SSS with patients' expectations as covariate (with error bars 95% confidence interval). The covariate patients' expectations is evaluated at the value 6.57 (adjusted mean).

Next we analyzed IBS-SSS at the various time points as within-subjects variable, with treatment as between-subjects factor and patients' expectations for their therapies as covariate (Figure 2). The assumptions for this repeated measure test are fulfilled (Box's test of equality of covariance matrices: *p* = 0.103; Levene's test of equality of error variances across treatment groups: *p* > 0.05 for all 4 time points).

A test for within-subjects effects showed, that there was no significant difference between Ayurveda and conventional nutritional therapy over time how they affected IBS-SSS [*p* = 0.281; *F*_(df1,error66)_ = 1.2; partial η^2^ = 0.018]. There was also no significant difference between patients' expectations of their therapy over time how they affected IBS-SSS (*p* = 0.130; *F* = 2.3; partial η^2^ = 0.034]. We had a nearly significant change in IBS-SSS over time (Wilks' lambda *p* = 0.050). There was the same change in mean IBS-SSS over time for both therapies (Wilks' lambda *p* = 0.548, i.e., there is no significant interaction).

Combining both treatments with patients' expectations for their therapies as covariate we obtain the following differences of estimated marginal means for IBS-SSS (Table 2).

**Table 2:** Pairwise comparison based on estimated marginal means.

**(I) IBS-SSS**	**(J) IBS-SSS**	**Mean difference (I-J)**	**Std. error**	**Sig.** ^a^	**95% confidence interval for difference** ^a^	
					**Lower bound**	**Upper bound**
Baseline	1 month	74.6^*^	9.5	0.000	48.7	100.5
	3 months	109.1^*^	10.5	0.000	80.6	137.6
	6 months	92.9^*^	11.9	0.000	60.5	125.3

* *The mean difference is significant at the 0.05 level*.

a *Adjustment for multiple comparisons: Sidak*.

Pairwise comparisons based on estimated marginal showed a clinically [mean difference (I-J) > MID (50)] and statistically significant improvement for all 3 time points compared to baseline (*p* < 0.001). In particular after 6 months there is still a significant positive effect for both treatments.

**Figure 3:**
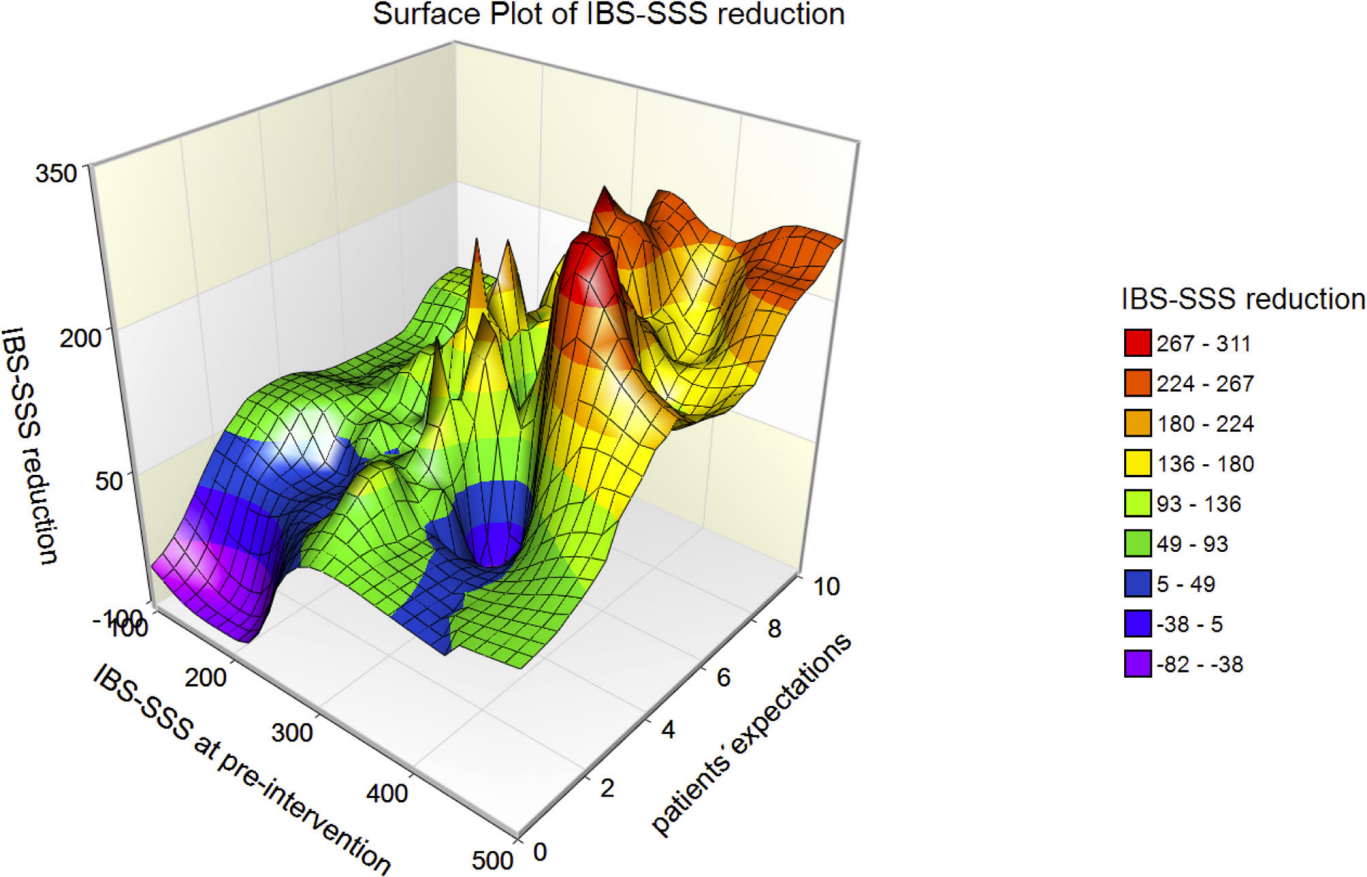
3D-surface plot of IBS-SSS reduction with respect to IBS-SSS at pre-intervention and patients' expectations. The higher IBS-SSS at pre-intervention and the larger the patients' expectations, the greater the IBS-SSS reduction.

The 3D-surface plot of IBS-SSS reduction (Figure 3) shows that the reduction increases with IBS-SSS at pre-intervention and with patients' expectations regarding treatment. Patients with expectations ≤ 4 had a mean IBS-SSS reduction of 38.8 ± 74.9, a value below the minimally clinically important difference (MID) of IBS-SSS (MID = 50).

Fifty percent of the participants with severe IBS changed to moderate and 30% to mild (Table 3).

**Table 3:** Crosstabulation of IBS severity classes by IBS severity classes after 3 months.

		**IBS-SSS after 3 months**		
		**Mild**	**Moderate**	**Severe**
IBS-SSS at pre-intervention	Mild	100.0%		
	Moderate	56.4%	41.0%	2.6%
	Severe	30.0%	50.0%	20.0%
Total		49.2%	42.6%	8.2%

*IBS-SSS, Irritable Bowel Syndrome-Severity Scoring System*.

### Secondary Outcomes

After 3 months, the mean values of the quality of life scores IBS-QOL were significantly (*p* < 0.001) improved from 53.4 ± 19.9 to 70.9 ± 20.9 in the Ayurveda group as well as significantly (*p* = 0.002) improved from 52.6 ± 21.8 to 64.5 ± 18.9 in the conventional group. The estimated marginal means adjusted for IBS-QOL at baseline and for participants' expectations at baseline as well as for study centers as a random effect were 15.8 ± 3.2 (95% CI = 9.4–22.3) for Ayurveda and 10.2 ± 3.5 (95% CI = 3.2–17.2) for conventional therapy. The mean difference of the improvement of IBS-QOL between baseline and 3 months based on estimated marginal means between both therapy groups was 5.5 ± 4.3 (95% CI = −3.1–14.1; *p* = 0.206). Sixty-three percent of the variance in IBS-QOL improvement after 3 months can be explained by the study treatments, 23.7% by IBS-QOL at pre-intervention and 3.2% by participants' expectations for their therapies at pre-intervention.

Table 4 is a summary of the most important results for the various outcomes. There were significant outcome improvements for most secondary outcomes treated with Ayurveda except for HADS-D (*p* = 0.070) and CPSS (*p* = 0.093) in contrast to conventional therapy, where all secondary outcome improvements were not significant except for the IBS-QOL score (*p* = 0.002) and the WHO-5 well-being index (*p* = 0.042).

**Table 4:** The means of the paired differences between outcome at baseline and after 3 months (paired samples test) and the effect of treatment (test of between-subjects effects).

**Outcome**	**Ayurveda**		**Conventional**		**Tests of between-subjects** **effects (treatment)**	
	**Mean of the** **paired differences ± std**	**Sig**	**Mean of the** **paired differences ± std**	**Sig**	**Sig**	**Partial η** ^**2**^
IBS - Severity Scoring System (IBS-SSS)	130.8 ± 81.2	<0.001	87.1 ± 99.5	<0.001	0.34	0.681
IBS - Quality Of Life (IBS-QOL)	17.5 ± 15.2	<0.001	11.9 ± 20.4	0.002	0.27	0.631
Cohen Perceived Stress Scale (CPSS)	1.9 ± 6.5	0.093	1.0 ± 5.6	0.289	0.62	0.281
Hospital Anxiety and Depression Scale (HADS-Total)	2.8 ± 6.1	0.01	−0.1 ± 5.2	0.947	0.41	0.582
Hospital Anxiety Scale (HADS-A)	1.8 ± 3.9	0.01	−0.3 ± 3.3	0.647	0.23	0.705
Hospital Depression Scale (HADS-D)	1.0 ± 3.3	0.07	0.2 ± 2.7	0.66	0.54	0.407
WHO-5 Well-Being Index	10.5 ± 21.3	0.006	6.7 ± 18.5	0.042	0.67	0.235

### Evaluation and Additional Questions

Ayurvedic intervention was rated slightly better than conventional intervention by the patients in several additional questions at the 3 months visit and the evaluation questions at the 6 months follow-up (Supplementary Tables in the Supplementary Material).

### Safety

There were 20 adverse events (12 in the Ayurveda group and 8 in the conventional group) throughout the intervention period in 19 participants (*n* = 11 in the Ayurveda group and *n* = 8 in the conventional group). No serious adverse event occurred. Adverse events were especially common cold (*n* = 8). Four events were possibly related to change of diet [2 in conventional group (obstipation; diarrhea) and 2 Ayurveda group (pyrosis; obstipation)].

### Adherence

The dieticians rated the adherence (external assessment based on dietary protocols and records made during the consultations) in the conventional group 8.0 ± 1.3 and in Ayurveda 7.0 ± 1.9 on NRS (0: not at all adherent to 10: very adherent). Self-rated compliance among the participants at the 3 months visit in the conventional therapy was 7.4 ± 1.5 and in Ayurveda 8.1 ± 1.5 and at the 6 months follow-up in the conventional therapy 6.5 ± 1.9 and in Ayurveda 6.2 ± 2.3 (0: not at all adherent to 10: very adherent).

## Discussion

The aim of this open-label multicenter randomized controlled clinical study with IBS patients was to investigate potential effects of Ayurvedic nutritional IBS therapy compared to conventional IBS therapy according to the recommendations of the German Nutrition Society including the low-FODMAP diet. The main findings of this study are:

- Both Ayurvedic and conventional therapy significantly reduced the primary outcome IBS-SSS at all 3 time points.
- The mean difference (51.1 ± 23.7) of the IBS-SSS reductions after 3 months between both therapy groups based on the estimated marginal means was statistically and clinically significant in favor of the Ayurveda group.
- Both therapies are equivalent in their contribution to the outcome variance.
- 68% of the variance in IBS-SSS reduction after 3 months can be explained by treatment, 6.5% by patients' expectations for their therapies and 23.4% by IBS-SSS at pre-intervention.
- Both patients' expectations of their therapies and the IBS-SSS at pre-intervention have a significant impact on the outcome. Notably, the higher the IBS-SSS score at pre-intervention and the larger the patients' expectations, the greater the IBS-SSS reduction.
- There were significant outcome improvements for all secondary outcomes in the Ayurveda group except for HADS-D, notably in contrast to conventional therapy where all secondary outcome improvements were not significant except for the IBS-QOL score and WHO-5.
- The compliance (both self-rated and externally assessed by dieticians) to the nutritional advices was high in both groups and deteriorated at the 6 months follow-up. Ayurveda was rated slightly better in the participant evaluation and additional questions. No serious adverse event occurred.

Preconceived expectations of treatment may cause IBS patients to perceive and record the results of their symptoms differently, a particular problem with IBS, where treatment results are subjective, very sensitive to individual behavior and a considerable placebo/unspecific effect (24). Among IBS study patients, the placebo response rate is high (25, 26). A meta-analysis showed a pooled estimate of the placebo response rate of 42.6% (95% CI = 38.0, 46.5) in CIM trials (25). Therefore, recent placebo-controlled trials provide robust evidence of clinical efficacy vs. placebo, and the first meta-analysis of low-FODMAP RCTs reported a greater likelihood of reducing abdominal pain (OR 1.81), abdominal bloating (OR 1.75), and general gastrointestinal symptoms (OR 1.81) compared to controls (27). In line with these findings, the German Nutrition Society (as well as nutritional societies of several other countries) now recommend a low-FODMAP diet to be considered if basic nutritional advices have been unsuccessful for the dietary management of IBS (21, 28).

Over the past decade, numerous studies have been published on the effectiveness of the low-FODMAP diet for reducing IBS symptoms. A 2017 review found that at least 10 randomized clinical trials had examined the effectiveness of the low-FODMAP diet during the short-term food-elimination phase, with 50–80% of participants reporting an improvement in IBS symptoms (29). In a systematic review and meta-analysis published in 2018, 9 studies with a total of 596 participants were examined in which a low-FODMAP diet was compared with various control diets. The low-FODMAP diet improved the symptoms of IBS compared with other diets with regard to gastrointestinal symptoms, abdominal pain, and health-related quality of life (30). In comparison to other low-FODMAP studies our conventional nutritional diet intervention including the low-FODMAP diet had lower improvement on the IBS-SSS.

The authors are not aware of other studies assessing Ayurveda nutrition therapy for IBS patients to date when this manuscript was written. Studies on Ayurvedic therapy modalities for IBS patients thus far have analyzed herbal interventions only and do not investigate the dietary aspects of Ayurvedic treatment approach in IBS (31, 32). The striking lack of scientific evidence here is in marked contrast to the central importance of nutrition in Traditional Indian Medicine Ayurveda in general, but especially in relation to nutrition-associated diseases.

In conjunction with the positive clinical effects, recent studies have also shown that the low-FODMAP diet, which is not easy to adhere to for many patients due to its restrictive choices of foods (6), can lead to profound and possibly detrimental changes in the microbiota and metabolome, the duration and clinical relevance of which are not yet known (29). Looking at comparable effects in both groups in the adjusted model for the main outcome, the Ayurvedic nutritional concept for IBS, which was well-tolerated and evaluated by most patients, might be an effective and comparatively “easy-to-adhere-to” alternative (or add-on) to the low-FODMAP concept. Subsequent studies are warranted particularly in this area to further illuminate the potential role of Ayurvedic nutrition for IBS patients and in other diseases of digestive dysfunction. Since Ayurveda is one of the two largest traditional medicine systems globally, along with TCM, and is increasingly being offered and used outside of its countries of origin, there is an obvious research gap (33). This gap should be filled by robust clinical studies using Whole Systems and Mixed-Methods research, among others, to find out whether such Ayurvedic concepts are effective, safe and implementable under Western conditions (34, 35). According to our interpretation of the Ayurvedic principles, IBS symptoms can be understood as an expression of “over-burdening” of the “digestive fire” agni. And our main hypothesis in this study was that any factor, which reduces the workload of agni and stabilizes its function, is helpful to reduce IBS symptoms. This approach we framed as general nutritional therapy of Ayurveda. In addition, symptom specific advice was given to each patient (specific nutritional therapy). The general therapeutic approaches could be comparable with a foundation, on which specific nutritional interventions can exert their action more effectively. The main general measures selected in this study in order to “deburden” agni were 1. warm food, 2. regular timings of meals which correlate with Ayurveda concepts of biorhythm, and 3. food articles which are generally light in digestion, but still satisfying and nourishing. The Supplementary Material *General and Specific Nutritional Therapy of Ayurveda* provides more details. According to the patients' life circumstance and their grade of motivation these ideals were individually adjusted during the nutrition consultations, which could lead to a partially reduced therapeutic effect.

The strengths of our study include the use of recommended and validated assessment tools and outcome parameters, clearly defined inclusion/exclusion criteria and consensual interventions in a multicentric setting. The implementation of an Ayurvedic nutritional approach that was both consequently based on traditional Ayurvedic paradigms and was adapted to a Western setting is also a strength of this study. Notably, only few minor adverse events occurred, suggesting that both interventions can be considered as safe and well tolerable which is of particular interest regarding the Ayurvedic concept since it had not been analyzed in a comparable setting before.

This study also has a number of limitations. First, the extent to which the observed effects were non-specific, particularly due to the attention of nutritional therapists, the influence of the specific settings and individual participants' beliefs and preconceptions about potential health effects of Ayurveda and meaning-responses cannot be estimated (25, 36). Second, this study did not have a minimal treatment or waiting list control group, thus the absolute effects of both of the interventions could not be calculated. Studies with a waiting control group or a participant preference trial are necessary to estimate non-specific effects (37, 38). Also, treatment effects might be linked to the natural course of the disease and/or patients might have variable symptoms. Third, a possible selection bias could not be excluded, as the majority of study participants was recruited via the Charité outpatient department for Complementary and Integrative Medicine. Fourth, the external physician could be a medical doctor of any specialty, so that a highly reliable IBS diagnosis such as one made by a board-certified gastroenterologist may not be guaranteed. Also, we used the national German S3 guidelines, which slightly differs from the IBS definition of the Rome III and IV consensus. Fifth, the drop-out rate in the conventional group was higher than anticipated and this poses another limitation to this study. The high attrition rate may introduce a bias into the results as we cannot rule out that participants dropped out due to dissatisfaction with or perceived ineffectiveness of the study intervention. Other reasons for drop-out may be related to the randomization to the different study interventions and the associated dissatisfaction of some participants. Also, a number of participants may have expected a faster relief through the interventions and thus may have experienced a loss of motivation when a relief of IBS symptoms did not emerge within the first weeks. Sixth, possible long-term effects remain unclear, as the study did not have long-term follow-ups. Seventh, this trial analysis was not analyzed by blinded statisticians. Eighth, this trial used the IBS-SSS as primary outcome. Alternatively, an 11-point NRS assessing worst abdominal pain in the past 24 h may also be adequate as a primary end point (39).

The conventional nutritional diet intervention including the low-FODMAP diet had lower improvement on the IBS-SSS in comparison to other low-FODMAP studies. This may indicate that our conventional nutritional counseling may not have provided advice strictly according to the low-FODMAP guidelines. The intervention was designed to explain the principles of a balanced diet for IBS patients in accordance with the German Nutrition Society and the essence of a low-FODMAP diet. We used dietary protocols as quality control and had them assessed by the consultants. However, we did not evaluate them from a nutritional point of view, so we do not know in detail if and to what extent the participants reduced the FODMAP content.

Another question to be addressed could be the feasibility and communicability of Ayurvedic dietary recommendations for IBS by conventional healthcare professionals without prior Ayurvedic knowledge. For example, as shown in the Supplementary Material of this publication, Ayurvedic dietary recommendations do not differ significantly in complexity from most other dietary recommendations for IBS, so that most likely no disproportionate effort would be required on the part of the relevant professionals to acquire the appropriate training and expertise. However, further transdisciplinary research would be desirable in this area as well.

## Conclusion

Patients with IBS seem to benefit significantly from both Ayurvedic and conventional nutritional counseling. The expectations regarding interventions influenced the outcome parameters. Based on these results, Ayurvedic nutrition therapy could be a useful part of IBS treatment. Multicenter confirmatory studies with higher patient numbers, longer-term follow-ups and patient preference designs are indicated to confirm the results of this study, also to clarify whether such a therapy might not be even more effective than established conventional nutritional therapies for the treatment of IBS.

## Data Availability Statement

The raw data supporting the conclusions of this article will be made available by the authors, without undue reservation.

## Ethics Statement

The studies involving human participants were reviewed and approved by Ethikkommission der Charité – Universitätsmedizin Berlin. The patients/participants provided their written informed consent to participate in this study.

## Author Contributions

MJ: guarantor of article, conceptualization, methodology, investigation, data curation, writing—original draft preparation, and project administration. TW: investigation, data curation, and writing—review and editing. DS, MM, DK-L, HC, and VM: writing—review and editing. LP: data curation, writing—review and editing. AM: conceptualization, supervision, and project administration writing—review and editing. NS: data curation and writing—review and editing. ES: writing—original draft preparation and review and editing. MW: formal analysis, data curation, data analysis, supervision, writing—original draft preparation, and review and editing. CK: conceptualization, methodology, supervision, project administration, and writing—review and editing. All authors approved the final version of the article.

## Conflict of Interest

CK and ES participate in training courses on Ayurveda at the European Academy for Ayurveda, Birstein and at Sonne und Mond, Berlin. MM participates in training courses on Ayurveda at the European Academy of Ayurveda, Birstein. The remaining authors declare that the research was conducted in the absence of any commercial or financial relationships that could be construed as a potential conflict of interest.

## Publisher's Note

All claims expressed in this article are solely those of the authors and do not necessarily represent those of their affiliated organizations, or those of the publisher, the editors and the reviewers. Any product that may be evaluated in this article, or claim that may be made by its manufacturer, is not guaranteed or endorsed by the publisher.
