# Supplementary material for: Ayurvedic vs. Conventional Nutritional Therapy Including Low-FODMAP Diet for Patients With Irritable Bowel Syndrome—A Randomized Controlled Trial
Source: Front Med (Lausanne). 2021 Sep 6;8:622029. doi: 10.3389/fmed.2021.622029 (PMC8450363; doi:10.3389/fmed.2021.622029)
Supplement: Supplementary file 1 [file Data_Sheet_1.pdf]

Supplemental Material: General and Specific Nutritional Therapy of Ayurveda

Refined over many centuries, nutritional therapy in Ayurveda Medicine is characterized by a high grade of complexity. As a personalized approach a multitude of aspects are discussed in Ayurveda texts[1] and are taken into account by Ayurveda practitioners in relation to ideal nutrition habits and food articles in each given case.[2,3]

The basic concept behind the Ayurveda intervention of this study concerning IBS is centered around the principle of *agni*, which can be conceived of as the 'strength' or capacity within the human organism to digest and metabolize food. To relate the *agni*-concept to modern nutritional physiology is challenging, since it is not rooted in the paradigm of molecular biology, but is an experience-based, rather dynamic functional principle, which cannot be limited to material entities, as for example enzymes.

According to our interpretation of the Ayurvedic principles, IBS symptoms can be understood as an expression of 'over-burdening' of the 'digestive fire' *agni*.[4] And our main hypothesis in this study was that any factor, which reduces the workload of *agni* and stabilizes its function, is helpful to reduce IBS symptoms. This approach we framed as *general nutritional therapy* of Ayurveda. In addition, symptom specific advice were given to each patient (*specific nutritional therapy*). The general therapeutic approaches could be comparable with a foundation, on which specific nutritional interventions can exert their action more effectively.

The main general measures selected in this study in order to 'deburden' *agni* were 1. warm food, 2. regular timings of meals which correlate with Ayurveda concepts of biorhythm, and 3. food articles which are generally light in digestion, but still satisfying and nourishing.[5] Table 1 provides practical details of all three factors. According to the patients' life circumstance and their grade of motivation these ideals were individually adjusted during the nutrition consultations, which could lead to a partially reduced therapeutic effect.

The *specific nutritional therapy* stems from a multitude of sources in the Ayurveda literature, taking into account Ayurveda specific nosological entities, concepts of pathophysiology and nutrition-pharmacological aspects.[6,7] Since the key complaints of IBS patients are varying from bloating, flatulence, abdominal pain and cramps to

---

[1] vide e.g. *Caraka Samhita*, in *Vimānasthāna* 1. R.K. Sharma and V. Bhagwan Dash, Editors. 2009, Chowkhamba Sanskrit Series.

[2] Gupta SN and Stapelfeldt E, *Ayurveda-Medizin: kāya-cikitsā - Therapiekonzepte für innere Erkrankungen* 2019: Thieme.

[3] Gupta, HH, *Āyurveda vijñāna* Band III: *svasthavṛtta-vijñāna* - āyurvedische Ernährungs- und Verhaltensmedizin 2017: Verlag für vedische Wissenschaften.

[4] *Caraka Samhita*, *Cikitsāsthāna* 15.42-44. R.K. Sharma and V. Bhagwan Dash, Editors. 2009, Chowkhamba Sanskrit Series.

[5] *Caraka Samhita*, in *Sūtrasthāna* 5.1-14 & 27.3 and *Vimānasthāna* 1.24f. & 2.1-9 and *Cikitsā-sthāna* 15.235-243. R.K. Sharma and V. Bhagwan Dash, Editors. 2009, Chowkhamba Sanskrit Series.

[6] vide e.g. *Caraka Samhita*, in *Sūtrasthāna* 25.38-40, 26.42-84 & 27 and *Cikitsāsthāna* 15 & 19. R.K. Sharma and V. Bhagwan Dash, Editors. 2009, Chowkhamba Sanskrit Series.

[7] Gupta SN and Stapelfeldt E, *Ayurveda-Medizin: kāya-cikitsā - Therapiekonzepte für innere Erkrankungen* 2019: Thieme.

constipation and diarrhea, for each condition specific individual advice were selected by the experienced Ayurveda nutrition consultant (table 1). According to Ayurvedic understanding, the first four symptoms are based on a similar pathophysiology and were therefore treated with similar measures.

Table 1: General and Specific Nutritional Therapy of Ayurveda

<table>
<tr><th colspan="3">GENERAL nutritional therapy of Ayurveda</th></tr>
<tr><td></td><td>Dos</td><td>Don'ts</td></tr>
<tr><td>warm</td><td>• 2-3 warm meals per day
• fruits (according to Ayurveda fruits don't have to be boiled)</td><td>• bread
• raw vegetables
• food or beverages directly from the fridge or refrigerator</td></tr>
<tr><td>regular</td><td>• <u>breakfast</u>: light (porridge with nuts & fruit or skipping breakfast)
• <u>lunch</u>: as the main meal of the day with more intensely nourishing food items
• <u>dinner</u>: early (between 6 & 7 p.m.)</td><td>• irregular meals
• snacking
• eating late at night</td></tr>
<tr><td>light in digestion, but well nourishing</td><td>• the amount of food shouldn't exceed the individual's capacity of digestion (agni)
• food articles containing higher amounts of <u>plant proteins</u> (e.g. nuts, almonds, seeds; mung beans & mung lentils, tofu, tempeh, seitan, full grain cereals)
• <u>specific fats</u> (especially olive oil, sesame oil, ghee (clarified butter)</td><td>• non-vegetarian (sausage, meat, fish)
• fatty or deep-fried foods
• ice cream
• bakery items
• cheese
• yoghurt (especially in the evening)</td></tr>
<tr><td>high quality & fresh</td><td>• preferably organic or / and home grown
• as often as possible freshly prepared</td><td>• junk food (burger, pizza), canned food
• highly processed food (e.g. cheese, savoury snacks, pies, pastry, sausage, ready meals)
• commercial soft drinks</td></tr>
<tr><td colspan="3"></td></tr>
<tr><th colspan="3">SPECIFIC nutritional therapy of Ayurveda</th></tr>
<tr><td>specific symptoms</td><td>Dos</td><td>Don'ts</td></tr>
<tr><td>bloating, abdominal pain, cramps</td><td>• carminative herbs & spices (e.g. fennel-, anise-, cumin-seeds, ginger, cardamom, asafoetida) half a glass (approx. 50-100 ml) of hot water to be sipped during meals
• "chew well and eat slowly" (especially carbohydrates)</td><td>• sweets (especially those containing white sugar)
• bakery items (bread, cakes, cookies - especially made from white flour)
• pasta made from white flour
• fast eating
• yeast containing food
• carbonated beverages
• most lentils and beans
• leafy cabbage
• garlic
• raw onions, bell pepper, cucumber
• leafy salads</td></tr>
<tr><td>constipation</td><td>• larger amounts (up to 20-40ml daily for 2-4 weeks) of specific fats, especially ghee (clarified</td><td>• goat's milk
• yoghurt especially from goat's milk
• "drying" food articles (e.g. coffee, black and</td></tr>
</table>

	butter), sesame oil, olive oil<br>• grape<br>• boiled, warm cow's milk<br>• spices<br>• dried fruit (e.g. plums, figs, raisins)<br>• drinking warm water and herbal teas throughout the day	green tea, millet, many pulses, puffed rice)<br>• nutmeg<br>• astringent fruit: banana, blueberries, papaya, pomegranate, sweet apples, persimmon
**diarrhea**	• cooked brown rice, puffed rice<br>• cooked red lentils<br>• astringent fruits: banana, blueberries, papaya, pomegranate, sweet apples, persimmon<br>• goat's milk (boiled)<br>• (goat's) yoghurt (only at lunch diluted with water, skimmed)<br>• nutmeg<br>• pumpkin, zucchini, fennel	• sour food (e.g. vinegar, wine, citrus fruit, pineapple, kiwi)<br>• spicy food (e.g. chili, ginger, garlic, horseradish, pepper)<br>• deep fried items<br>• grapes, dried fruit<br>• cow's milk<br>• alcohol
