# Supplementary material for: Ayurvedic vs. Conventional Nutritional Therapy Including Low-FODMAP Diet for Patients With Irritable Bowel Syndrome—A Randomized Controlled Trial
Source: Front Med (Lausanne). 2021 Sep 6;8:622029. doi: 10.3389/fmed.2021.622029 (PMC8450363; doi:10.3389/fmed.2021.622029)
Supplement: Supplementary file 2 [file Table_1.PDF]

Supplemental Material: Evaluation and Additional Questions

Additional questions at 3 months visit [1: agree fully, 2: agree, 3: neither, 4: disagree, 5: disagree fully]

	Conventional	Ayurveda
1. The treatment has significantly reduced my pain	2.7±1.0	1.8±0.7
2. The treatment has significantly reduced my complaints	2.6±1.0	1.8±0.8
3. The treatment has significantly improved my quality of life	2.6±1.0	2.1±0.9
4. I have tolerated the change of diet well	1.7±0.7	1.5±0.8
5. I am satisfied with the nutritional advice	1.5±0.8	1.3±0.7
6. The duration of the nutritional counselling was appropriate	1.6±0.7	1.6±0.8
7. The change in diet was easy for me to integrate into everyday life	2.0±0.8	2.6±1.0
8. I can imagine bearing the costs of the nutritional advice myself	2.6±1.1	2.6±1.2
9. The participation in the study was worthwhile for me	2.0±1.2	1.3±0.5
10. The ratio of treatment effort to benefit is low	3.0±1.1	3.0±1.5
11. I can imagine continuing to eat as suggested in the study	1.6±0.5	1.4±0.5
12. I am satisfied with the result	2.5±1.0	1.7±0.8

Evaluation questions at 6 months follow-up [NRS - 0: not at all to 10: very]

	Conventional	Ayurveda
1. Did you find the nutritional counselling to be generally helpful for you?	6.3±2.8	8.8±1.5
2. Did the nutritional counseling help you with your life?	5.8±2.6	8.1±2.5
3. Would you recommend the nutritional advice to your friends and family?	6.8±2.9	8.6±2.3
4. How much do you want to continue to implement the nutritional counselling?	7.2±2.3	8.0±2.7
5. Did you eat a warm diet? [Ayurveda only]	-	7.5±2.1
6. Have you been eating regularly? [Ayurveda only]	-	7.5±2.1
7. Have you been eating easily digestible food? [Ayurveda only]	-	7.9±1.8
8. Have you listened to your feelings of hunger and satiety and to which foods are easily digestible for you? [Conventional only]	6.8±2.0	-
9. Have you eaten a varied diet and also tested new foods for their digestibility from time to time? [Conventional only]	6.6±2.0	-
10. Have you made sure that you regularly take your meals "in peace" and chew well? [Conventional only]	6.3±2.0	-
